# Self-Cleaning Glass of Photocatalytic Anatase TiO_2_@Carbon Nanotubes Thin Film by Polymer-Assisted Approach

**DOI:** 10.1186/s11671-016-1674-4

**Published:** 2016-10-13

**Authors:** Qinghua Yi, Hao Wang, Shan Cong, Yingjie Cao, Yun Wang, Yinghui Sun, Yanhui Lou, Jie Zhao, Jiang Wu, Guifu Zou

**Affiliations:** 1 College of Physics, Optoelectronics and Energy and Collaborative Innovation Center of Suzhou Nano Science and Technology, Soochow University, Suzhou, 215006 People’s Republic of China; 2 Department of Electronic and Electrical Engineering, University College London, Torrington Place, London, UK

**Keywords:** CNTs, TiO_2_, Photocatalysis, Polymer-assisted approach, Self-cleaning

## Abstract

Due to the good photocatalytic activity, the TiO_2_@CNTs thin film is highly desirable to apply to the self-cleaning glass for green intelligent building. Here, the TiO_2_@CNTs thin film has been successfully achieved by polymer-assisted approach of an aqueous chemical solution method. The polymer, polyethylenimine, aims to combine the Ti^4+^ with CNTs for film formation of TiO_2_@CNTs. The resultant thin film was uniform, highly transparent, and super-hydrophilic. Owing to fast electron transport and effectively hindering electron-hole recombination, the TiO_2_@CNTs thin film has nearly twofold photocatalytic performance than pure TiO_2_. The TiO_2_@CNTs thin films show a good application for self-cleaning glasses.

**Electronic supplementary material:**

The online version of this article (doi:10.1186/s11671-016-1674-4) contains supplementary material, which is available to authorized users.

## Background

The serious environmental pollutions have attracted attention widely with the rapid growth of worldwide industry [1]. To tackle the pollution problem, photocatalysis has been demonstrated as a low-cost and sustainable technology for removing harmful organic contaminations in air and water. Since 1972 when Fujishima [2] discovered the photocatalytic activity on the titanium oxide (TiO_2_) photoanode in electrochemical cell under a UV-light, a great deal of semiconductor photocatalytic materials have been investigated [3, 4]. TiO_2_ has been a utility photocatalytic material owing to its low-cost, high oxidative efficiency, nontoxicity, and high chemical stability. However, the pure TiO_2_ has limited performance by easy recombination between the electrons and holes [5–8]. As it is well known, carbon nanotubes (CNTs) show good electron-accepting capability and fast electron transport [9–11]. Hoffmann proposed that TiO_2_@CNTs absorbed the energy and excited the electrons under illumination, and then, the electrons may transfer to CNTs while the holes may remain on TiO_2_ [12]. The electron and hole can be effectively separated by CNTs. Simultaneously, many research work have investigated the TiO_2_@CNTs system which indicated that the TiO_2_@CNT composite would be a promising candidate to enhance the photocatalytic performance [13–18]. Therefore, TiO_2_@CNTs is highly desirable to apply to the self-cleaning glass for green intelligent building. Usually, self-cleaning glass should require high photocatalytic activity, chemical stability, and super-hydrophilicity (super-hydrophobic) large area thin film [19, 20]. Up to now, Daeyeon Lee and coworkers fabricated the MWCNTs (multi-wall carbon nanotubes)/TiO_2_ thin films with significant enhancement photocatalytic performance through a layer-by-layer assembly method involving the MWCNTs incorporated TiO_2_ thin film [21]. Kim’s group prepared the CNT/TiO_2_ core/shell thin film and demonstrated the excellent performance [7] Oliveira’s group deposited the TiO_2_/MWCNTs coating with wettability and photocatalytic activity by sol-gel method [22]. Here, we design an approach to deposit TiO_2_@CNTs thin films with excellent photocatalytic performance for self-cleaning glass.

As well known, the surface of CNTs can be functionalized with hydrophilic groups (−COOH, −C = O, −OH) by acid oxidation [23, 24], and the hydrophilic group will react with the amine group of polyethylenimine (PEI) [25–27]. Meanwhile, Jia et al. reported that the Ti^4+^ could be combined with PEI to grow high-quality epitaxial TiO_2_ thin films [28, 29]. Inspired by these, we attempt to prepare Ti^4+^ combining PEI along with CNTs forming a stable and homogeneous precursor solution to fabricate TiO_2_@CNTs thin film. In general, most of work [22, 30] (such as O. Akhavan et al. and Dai et al.) reports sol-gel method, hydrolysis of titanium isopropoxide in CNTs solution, to deposit TiO_2_@CNTs thin film. Kwadwo E. Tettey group employed oppositely charged species of layer-by-layer (LbL) assembly approach to grow TiO_2_@CNTs thin film [21]. Muduli et al. utilized a hydrothermal method to prepare TiO_2_-MWCNTs composite [31]. Here, we provide a polymer-assisted deposition approach to grow TiO_2_@CNTs thin film. Polymer-assisted deposition approach has a key aqueous and homogenous system. The polymer can prevent ion hydrolysis in the solution, and the precursor could store in air stability for several months. The Ti ions and CNTs can be well distributed in precursor. The grown TiO_2_@CNT thin film shows the CNTs are homogenously embedded in the thin film. The viscosity and the concentration of the Ti^4+^ in precursor can be ready to tune the thickness of the thin films. This approach has the significant advantage over other methods, is a “bottom-up” method, and can form the conformal coating for complex structure. As a result, TiO_2_@CNTs thin film is readily grown with excellent photocatalytic activity. Both pure TiO_2_ thin film and TiO_2_@CNTs thin film have the high crystallinity and homogeneous surface. Owing to feasible design of carriers’ separation, the TiO_2_@CNTs thin films show better photocatalysis than the pure TiO_2_ thin films. It advances to deposit TiO_2_@CNTs in self-cleaning glasses by polymer-assisted approach.

## Methods

### Synthesis of CNTs-Ti^4+^ Precursor Solution

The Ti^4+^-PEI was prepared by an aqueous solution of Ti^4+^ ion bound to polymer. The solution containing 4 g of PEI and 4 g of ethylenediaminetetraacetic acid (EDTA) was stirred vigorously for a while until the PEI dissolved completely. Meanwhile, 2.5 g of TiCl_4_ was slowly added into a solution which included 5.0 g of 30 % H_2_O_2_ and 5 ml of water. Then, the TiCl_4_ solution was dropwise added to the PEI solution and maintained the pH of the PEI solution at about 7.5 by NH_3_·H_2_O until the mixture solution appears a precipitate. Finally, the solution was placed in an Amicon ultrafiltrations unit including an ultrafiltration membrane under 60-psi argon pressure. The Amicon ultrafiltration unit is designed to cut off <10,000 g/mol molecular weight while reserving the desired materials. The solution was diluted several times. The concentration of the final solution was 23.56 mg/ml which was conducted with inductively coupled plasma atomic emission spectroscopy (ICP-AES, PerkinElmer Optima 8000). CNTs grown by chemical vapor deposition (CVD) were purchased from Shenzhen Nanoport. Then, the CNTs was functionalized by refluxing in a mixture of concentrated nitric and sulfuric acids (1:3 in volume). The CNTs aqueous solution was added into Ti^4+^-precursor solution with stirring and vigorous ultrasonication for complete dispersion. We designed an experiment of thermal gravimetric analyzer (TGA) to characterize the final product (Additional file 1: Figure S1). The final product is matched with the ratios of the CNT added in the Ti^4+^ precursor at the beginning of the experiments. So, the ratio of CNTs in TiO_2_@CNT films is about 0, 2, 5, 8, and 10 %.

### Preparation of TiO_2_@CNTs

For the TiO_2_@CNTs films, the final precursor was spin-coated on quartz at 2500 rpm for 30 s, then the film was calcinated at 400 °C for 2 h in air. Similarly, a reference experiment in the absence of CNTs was prepared.

### Characterization

Ti^4+^ concentration in precursor solution was measured by inductively coupled plasma atomic emission spectrometer (ICP-AES, PerkinElmer Optima 8000). X-ray diffraction (XRD) measurement was taken on a Riguku D/MAX-2000PC diffraction system to evaluate the crystal structure. Raman shift spectroscopy was recorded using HR800 (Raman HORIBA Jobin Yvon) with a 514-nm excitation wavelength. Scanning electron microscopy (SEM) images were observed on SU8010 (HITACHI) systems. The ultraviolet-visible (UV-VIS) spectrum was tested by using Shimadzu UV-2450. The water contact angle of the film surfaces was carried on contact angle measurement (Data Physics OCA). The electrochemical impedance spectra were tested by electrochemical workstation.

### Photocatalytic Experiments

Methyl orange (MO) dye was used to study the photocatalytic activity of the samples under a UV-light irradiation (5 W). The concentration of the MO dye aqueous solution is 10 mg/L. The TiO_2_ thin films and TiO_2_@CNTs thin films were fastened by cotton and immersed in the MO (10 mg/L) aqueous solution, respectively. Before illumination, the MO dye aqueous solution was stirred for 1 h under dark for adsorption-desorption equilibrium. After that, the MO dye solution was irradiated under a UV-light. At a 20-min interval, the solution was analyzed by recording the UV-spectrum of MO dye aqueous solution.

## Results and Discussion

**Scheme 1:**
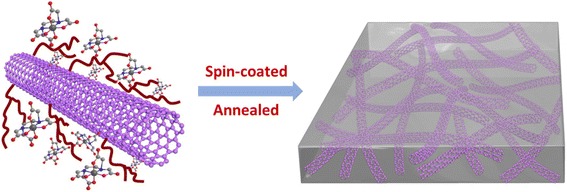
Schematic process for TiO_2_@CNTs

Scheme 1 depicts the preparation process of TiO_2_@CNTs thin film. The PEI combines to Ti^4+^ complex by static electricity forming the PEI-Ti^4+^ precursor solution and the functional CNTs bind with the amino group of PEI. Here, PEI acts as a “bridge” to link Ti^4+^ complex and CNTs to form the Ti^4+^ complex-CNTs precursor. Simultaneously, PEI can control the viscosity of the precursor solution to help film formation by spin-coating process.

**Fig. 1:**
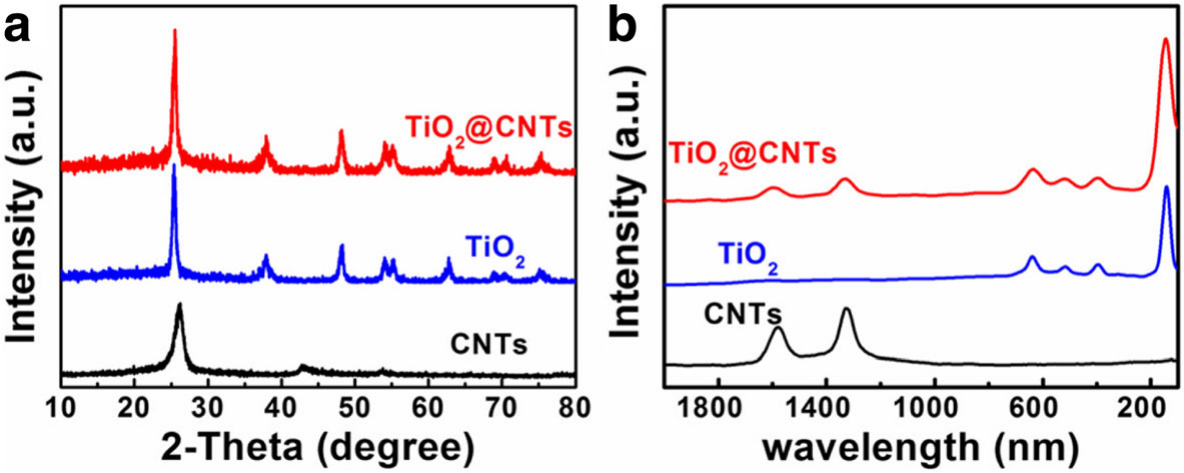
XRD patterns (**a**) and Raman spectrum (**b**) of CNTs, TiO_2_, and TiO_2_@CNTs, respectively

The products are analyzed by X-ray diffraction (XRD) measurement. As shown in Fig. 1a, there are two black peaks at 26.2° and 42.8° corresponding to (002) and (100) diffractions of CNTs. The peaks of XRD blue pattern at 25.38°, 37.94°, 48.3°, 54.04°, 55.16°, 62.72°, 68.9°, 70.38°, and 75.16° are indexing to (101), (004), (200), (105), (211), (204), (116), (220), and (215), which confirms to anatase TiO_2_ (JCPDS card No.21-1272). There are no any other peaks which indicate that the pure anatase TiO_2_ sample has been obtained. In the XRD red pattern, there is no obvious CNTs diffraction peak seen in TiO_2_@CNT thin film because of strong background and diffraction peak (101) overlap of TiO_2_ [32]. Raman microscopy shown in Fig. 1b is used to further analyze CNTs, TiO_2_, and TiO_2_@CNTs. The characteristic bands of black peaks at 1331 and 1595 cm^−1^ are indexing to D band and G band of CNTs. The blue peaks at 147, 397, 522, and 640 cm^−1^ are corresponding to E_g_, B_1g_, B_1g_, and E_g_ active modes of anatase TiO_2_. Seen from the red peaks of Raman spectra, there were not only the characteristic bands of anatase TiO_2_ but also the D band and G band of CNTs, confirming the presence of both anatase TiO_2_ and CNTs in the final of product TiO_2_@CNTs composite [33].

**Fig. 2:**
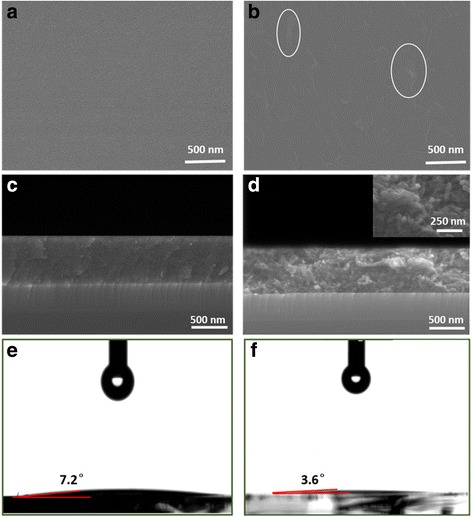
SEM images of as-prepared pure TiO_2_ thin film (**a**) and TiO_2_@CNTs thin film (The CNTs are labelled by ellipse, typically.) (**b**); the cross-sectional images of as-prepared pure TiO_2_ thin film (**c**) and TiO_2_@CNTs thin film (**d**); the images of water contact angle test for pure TiO_2_ thin film (**e**) and TiO_2_@CNTs thin film (**f**)

Self-cleaning performance of TiO_2_@CNTs thin films is achieved by super-hydrophilicity and good photocatalytic activity. The surface morphologies of the pure TiO_2_ film and TiO_2_@CNTs thin film are investigated by using a scanning electron microscope (SEM) as shown in Fig. 2a, b). The SEM images show that the thin films are homogeneously covered on the substrates. Seen from the TiO_2_@CNTs thin films, most of CNTs are embedded in TiO_2_ thin film and only few CNT is bared on the surface of the thin film. The cross-sectional images of pure TiO_2_ thin film and TiO_2_@CNTs thin film (Fig. 2c, d) show a clear interface between the substrate and the thin film, and the thickness of the thin films is about 500 nm. Meanwhile, the pure TiO_2_ thin film is stacked by nanoparticle while the TiO_2_@CNTs thin film is composed of nanoparticles along with the CNTs. The water contact angle is measured to analyze the wettability of the films. The contact angles of the pure TiO_2_ films and TiO_2_@CNTs thin film are 7.2° and 3.6° in Fig. 2e, f, respectively. Compared with the glass’s 40.7° (Additional file 1: Figure S3), both the TiO_2_ and TiO_2_@CNTs thin films’ contact angles are hydrophilic (<10°). The TiO_2_@CNTs thin films show super-hydrophilicity (<5°). Super-hydrophilic TiO_2_@CNTs thin film, preventing water condensation on a substrate, is of great importance for self-cleaning applications. It is noted that transmission of the thin films is another advanced property to self-cleaning glass. Additional file 1: Figure S2 illustrates the transparency of quartz, TiO_2_ thin film on quartz substrate, and TiO_2_@CNT thin film on quartz substrate. Although the TiO_2_@CNT thin film shows lower transparency than the TiO_2_ thin film, both of them have good transmittance.

**Fig. 3:**
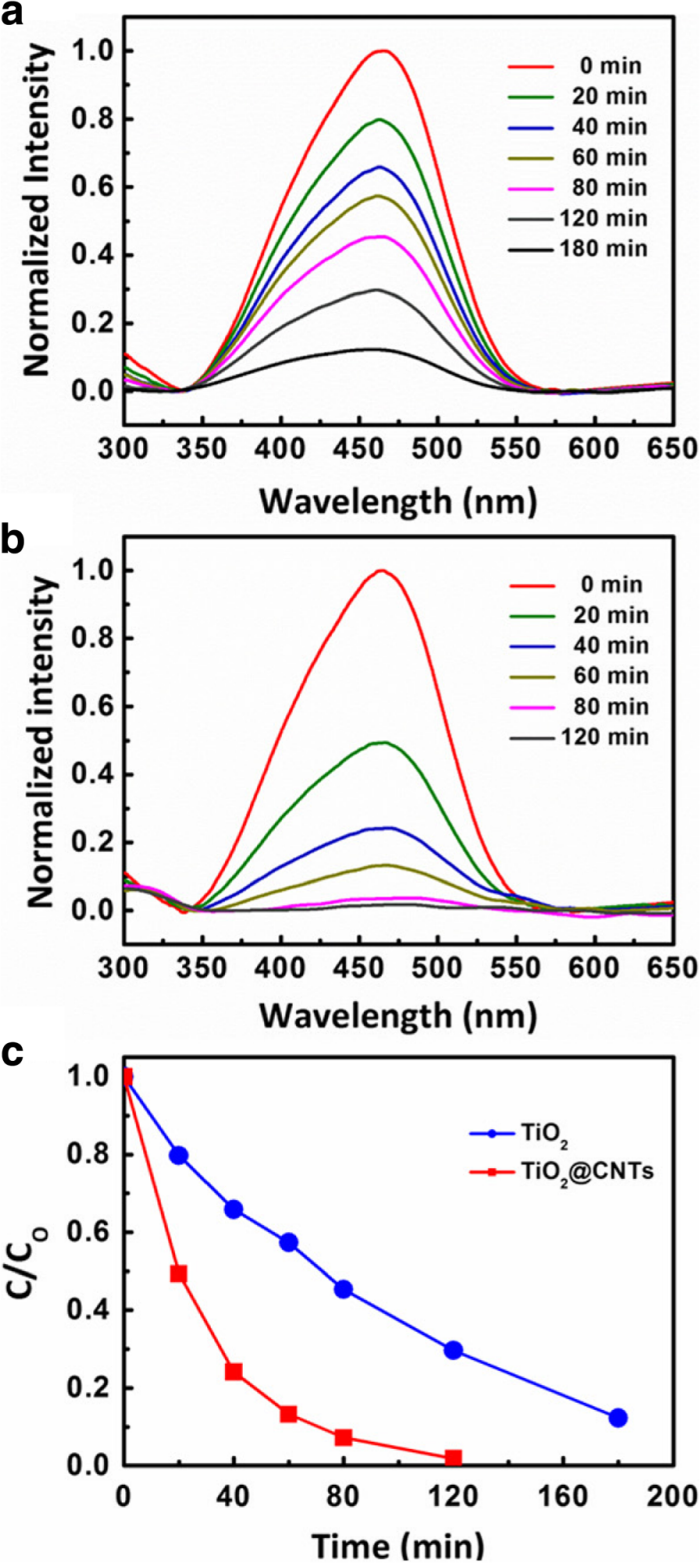
The UV-Vis absorption spectra of a MO dye solution under UV-irradiation with (**a**) pure TiO_2_ catalyst and (**b**) TiO_2_@CNTs composite catalyst. (**c**) Relative changes of the MO dye concentration as a function of reaction time with pure TiO_2_ catalyst and TiO_2_@CNTs composite catalyst

The photocatalytic activities of TiO_2_@CNTs thin film and pure TiO_2_ thin film are evaluated by methyl orange (MO) dye degradation under UV-light irradiation after stirring in the dark for adsorption-desorption equilibrium. The photocatalysis performance about different content CNTs in TiO_2_@CNTs thin film is shown in Additional file 1: Figure S4. The results show that the TiO_2_@CNTs thin film with 5 % CNTs has the best performance. For comparison, the photocatalytic performance of the pure TiO_2_ thin film and TiO_2_@CNTs (5 %) thin film is detailedly analyzed as follows. The absorption spectra of the MO dye aqueous solution are shown in Fig. 3a, b with increasing UV-light irradiation time. The absorption peak at 465 nm is the intrinsic absorption of MO dye. The absorption peaks of MO dye are decreased upon time with TiO_2_@CNTs composite catalyst and pure TiO_2_ catalyst. The absorption peak diminishes completely after 80 min for the TiO_2_@CNTs thin film. However, as for the pure TiO_2_, the absorption peak decreases slowly, and the absorption peak of dyes does not disappear even after 180 min. The gradual decrease in the absorption of the MO dye is attributed to the decomposition of MO dye [34, 35]. The degradation of MO dye is carried out by hydroxyl radicals which are generated by transferring electrons and holes from photocatalytic materials to the dye aqueous solution. In Fig. 3c, the normalized temporal concentration changes (*C*/*C*_0_) of MO dye aqueous solution can also illustrate the photocatalytic performance during the degradation (*C*_0_ is the initial concentration of MO dye and *C* is the concentration of MO dye with different reaction time). After 80-min UV-light irradiation, over 93 % and ~55 % of the initial dyes are decomposed by TiO_2_@CNTs composite and pure TiO_2_, respectively. The photocatalytic efficiency of TiO_2_@CNTs thin films is nearly twofold that of TiO_2_ thin film. Therefore, the results demonstrate that the photocatalytic performance was greatly enhanced by the TiO_2_@CNTs thin film. The performance is comparable to the others in the literature [5, 7]. Meanwhile, the TiO_2_@CNTs thin films show good stability and recyclability in Additional file 1: Figure S5.

**Fig. 4:**
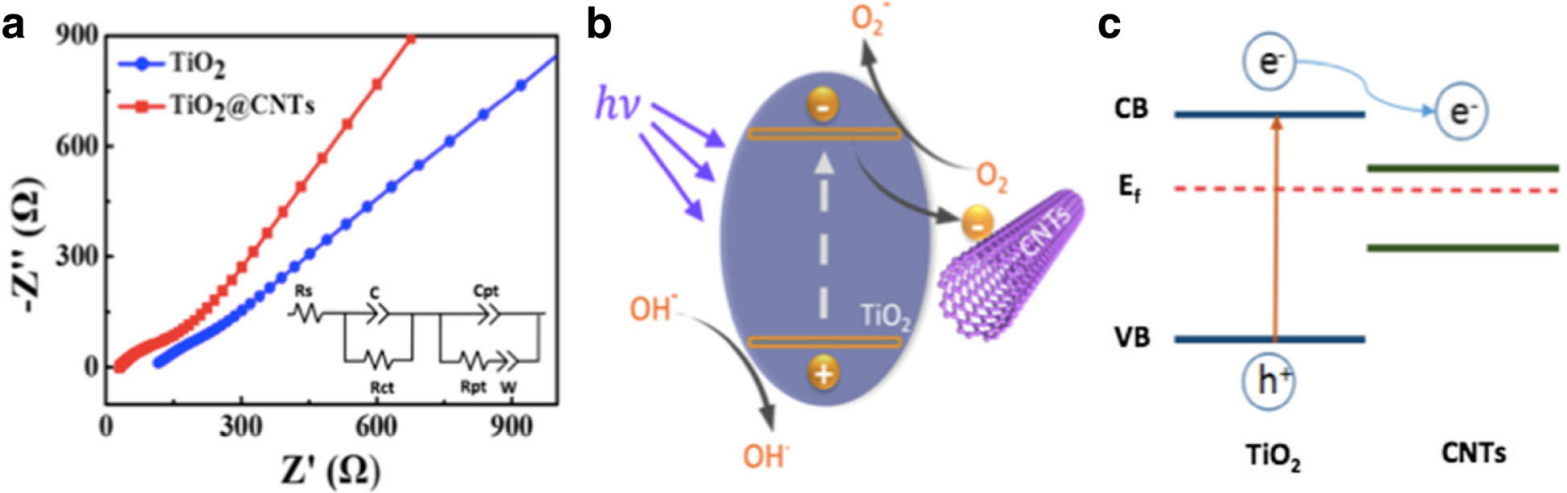
(**a**) The Nyquist plots of pure TiO_2_ and TiO_2_@CNT composite electrodes. (**b**) The schematic illustration of the charge transfer mechanism. (**c**) The energy band diagram of TiO_2_/CNTs structure

The photoelectric properties of pure TiO_2_ and TiO_2_@CNTs help to analyze the enhanced photocatalysis of TiO_2_@CNTs. The electrochemical impedance spectra can effectively illustrate the electron transport between the electrode and electrolyte interfaces. The equivalent electrical circuit mode can be drawn from Nyquist plot. The semicircle is attributable to the total charge transfer resistance (Rcf) and the capacitance of the space charge region (C) including charge transfer across the FTO/TiO_2_@CNTs interface and TiO_2_@CNTs/electrolyte interface [36, 37]. For TiO_2_@CNTs, Rcf and C are 53.1 Ω and 4.83 × 10^−7^ f, respectively. While for pure TiO_2_, Rcf and C are 320 Ω and 35.4 × 10^−6^ f, respectively. Based on the Nyquist plots (Fig. 4a), it shows a decrease in the interface layer resistance and charge transfer resistance on the TiO_2_@CNTs surface. The smaller radius of the arc in Nyquist plots means smaller resistance and faster electron transportation [38, 39]. In addition, the charge transfer mechanism between the TiO_2_ and CNTs during the photocatalysis is shown in Fig. 4b and the energy band diagram of TiO_2_/CNTs structure is shown in Fig. 4(c). Under the illumination, the electron (e^−^) of TiO_2_ are excited to the conductor band while the holes (h^+^) are left to the valence band for oxidation of MO dye. Meanwhile, the hole can adsorb the OH^−^ in the water and generates the OH ^.^, and the electron can react with the O_2_ to form the O_2_^−^. The O_2_^−^ and OH^.^ show strong degradation of dye. Furthermore, owing to the low Fermi level of CNTs, the electrons are easily injected to CNTs. Another important role of CNTs in the C composite is being a fast electron transporter due to its one-dimensional structure. As a result, the electron-accepting and electron-transporting properties of CNTs in TiO_2_@CNTs composite can effectively hinder electron-hole pair recombination and leave more holes to promote the degradation of MO dyes. This phenomenon has been demonstrated by a large number of previous dye degradation [15, 40, 41]. TiO_2_@CNTs thin films have high-quality, super-hydrophilic surface and good photocatalytic performance to stimulate its application in the fields of self-cleaning glass.

## Conclusions

In summary, we developed a polymer-assisted approach to synthesize the anatase TiO_2_@CNTs thin films. The polymer not only urges TiO_2_ and CNTs combination but also facilitates TiO_2_@CNTs film formation. The resultant TiO_2_@CNTs films are compact and present good transmission and super-hydrophilicity. Due to advancing fast electron transport and effectively hindering electron-hole pair recombination, TiO_2_@CNTs thin film performs the photocatalytic efficiency with nearly twofold to pure TiO_2_. The aqueous and feasible technology of thin film fabrication brings TiO_2_@CNT thin film to be a good candidate for self-cleaning glasses.

## Additional file

Additional file 1: Supporting information. (DOCX 860 kb)

## Abbreviations

CNTs: Carbon nanotubes
ICP-AES: Inductively coupled plasma atomic emission spectrometer
MO: Methyl orange
SEM: Scanning electron microscope
TiO_2_: Titanium oxide
XRD: X-ray diffraction
