# Supplementary material for: Self-Cleaning Glass of Photocatalytic Anatase TiO2@Carbon Nanotubes Thin Film by Polymer-Assisted Approach
Source: Nanoscale Res Lett. 2016 Oct 13;11:457. doi: 10.1186/s11671-016-1674-4 (PMC5063831; doi:10.1186/s11671-016-1674-4)
Supplement: Additional file 1: — Supporting information. (DOCX 860 kb) [file 11671_2016_1674_MOESM1_ESM.docx]

**Supporting Information for**

**Self-cleaning Glass of Photocatalytic Anatase TiO_2_@Carbon Nanotubes Thin Film by Polymer-assisted Approach**

Qinghua Yi,^1^ Hao Wang,^1^Shan Cong,^1^ Yingjie Cao,^1^ Yun Wang,^1^ Yinghui Sun,^1^ Yanhui Lou,^1^ Jie Zhao,^1^ Jiang Wu^2^ and Guifu Zou*^1^

^1^College of Physics, Optoelectronics and Energy & Collaborative Innovation Center of Suzhou Nano Science and Technology, Soochow University, Suzhou, 215006, P.R.C. China.

^2^Department of Electronic and Electrical Engineering University College London, Torrington Place, London, UK

*corresponding authors: [zouguifu@suda.edu.cn](mailto:zouguifu@suda.edu.cn)


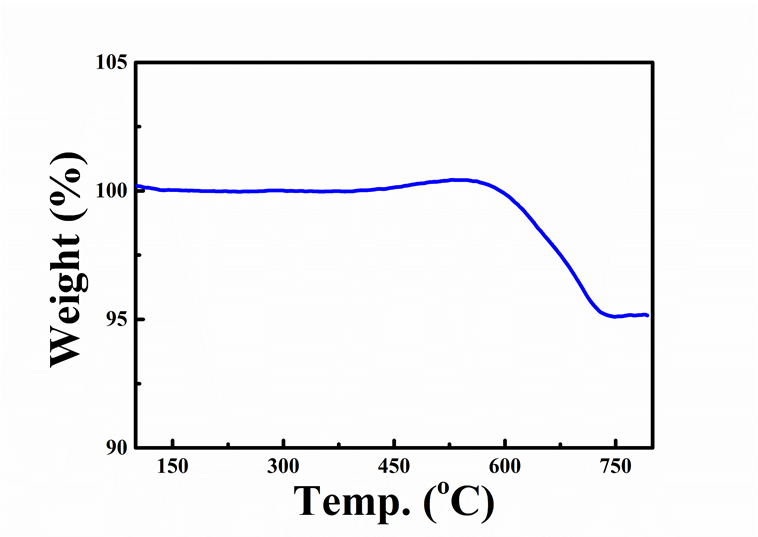


**Figure 1S** Thermal Gravimetric Analyzer (TGA) to characterize the CNTs content in final TiO_2_@CNTs product.


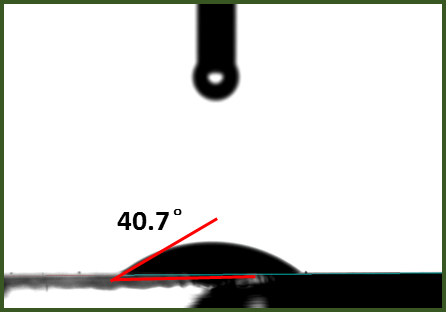


**Figure 2S** The contact angle images of glass.

**Figure 3S** The transparency of reference, quartz, TiO_2_ thin film on quartz and TiO_2_@CNTs thin film on quartz.

**
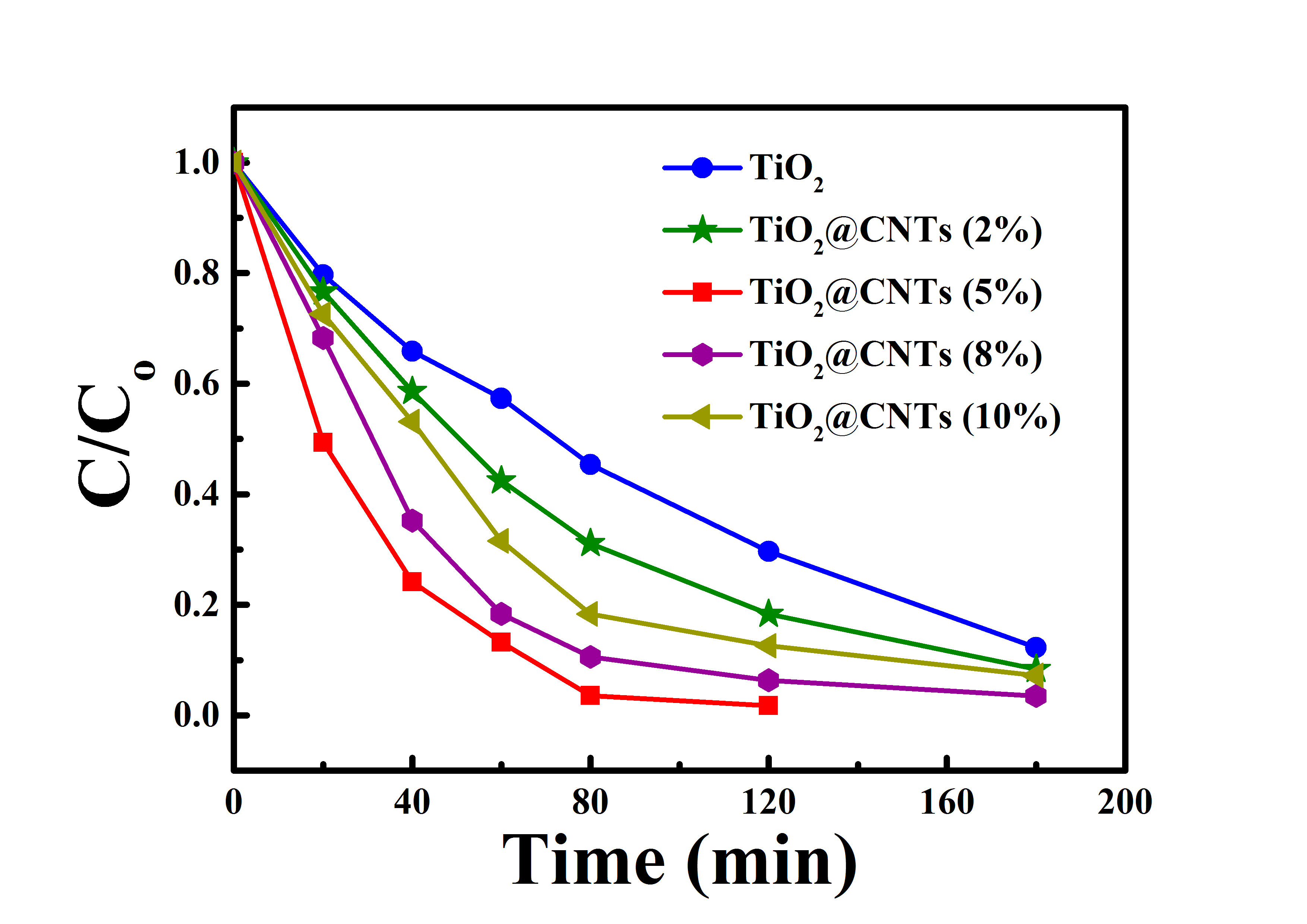
**

**Figure 4S** Relative changes of the MO dye concentration as a function of reaction time with TiO_2_@CNTs composite catalysts (CNTs content: 0%, 2%, 5%, 8%, 10%).


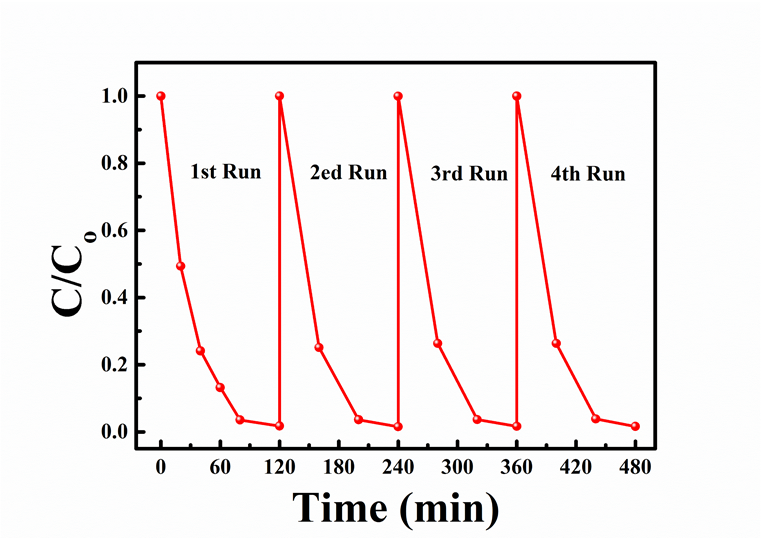


**Figure 5S** the stability and recyclability of the anatase TiO_2_@CNTs thin film are analyzed by repeating MO degradation under UV-light illumination four times.
